# Unequal Access to Evidence-Based Cardiometabolic Therapies: Racial Disparities in SGLT2 Inhibitor and GLP-1 Receptor Agonist Use Among Patients With Type 2 Diabetes and Cardiovascular Disease

**DOI:** 10.7759/cureus.106266

**Published:** 2026-04-01

**Authors:** Mukosolu F Obi, Shardil Ahmad, Kamsy Ezechukwu

**Affiliations:** 1 Cardiology, Ochsner Medical Center, New Orleans, USA; 2 Internal Medicine, The Brooklyn Hospital Center, Brooklyn, USA; 3 Internal Medicine, Louisiana State University Health Sciences Center, Baton Rouge, USA

**Keywords:** chronic heart failure, diabetes type 2, gender disparities, glp-1 receptor agonists, racial and ethnic disparities, sglt-2 inhibitor

## Abstract

The pharmacologic management of type 2 diabetes mellitus (T2DM) and cardiovascular diseases, including heart failure, has advanced significantly with the introduction of glucagon-like peptide-1 receptor agonists (GLP-1 RAs) and sodium-glucose co-transporter-2 inhibitors (SGLT2is), therapies that provide substantial cardiovascular and renal benefits. Despite robust evidence supporting their use, access to and utilization of these agents remain unevenly distributed across patient populations. This literature review explores disparities in the prescribing patterns of GLP-1 RAs and SGLT2is, with particular attention to the influence of race, ethnicity, gender, and socioeconomic status. By integrating findings from recent studies, this review aims to uncover the root causes of these disparities and outline approaches to ensure more equitable delivery of cardiometabolic care.

## Introduction and background

Type 2 diabetes mellitus (T2DM) is characterized by both insulin resistance and progressive β-cell dysfunction, leading to inadequate insulin secretion and chronic hyperglycemia. Its global prevalence has more than doubled in recent decades, driven largely by rising obesity rates and increasingly sedentary lifestyles. Beyond its metabolic burden, T2DM is a major contributor to cardiovascular disease and chronic kidney disease, which together account for substantial morbidity and mortality worldwide [1].

The emergence of glucagon-like peptide-1 receptor agonists (GLP-1 RAs) and sodium-glucose co-transporter-2 inhibitors (SGLT2is) has transformed the therapeutic landscape of T2DM. These agents not only improve glycemic control but also deliver robust cardiovascular and renal protection, with additional anti-inflammatory and antioxidative effects that modify the underlying pathophysiology of cardiometabolic disease [2,3]. Accordingly, major international guidelines now recommend GLP-1 RAs and SGLT2is for individuals with T2DM and established cardiovascular disease, heart failure, or chronic kidney disease, regardless of baseline glycemic control. Despite this strong evidence base, real-world prescribing patterns have not kept pace with clinical recommendations. The utilization of GLP-1 RAs and SGLT2is remains suboptimal overall, and critically, these therapies are not prescribed equitably. A consistent body of literature demonstrates that Black and Hispanic patients, women, and individuals with lower socioeconomic status are significantly less likely to receive these agents, even when they meet clear clinical criteria. These disparities persist across healthcare settings and are influenced by intersecting factors, including provider prescribing bias, structural barriers to specialty care, medication costs, and restrictive insurance policies [4,5].

In the United States, these inequities are particularly concerning, as the populations least likely to receive SGLT2 inhibitors and GLP-1 receptor agonists are often those at highest risk for adverse cardiometabolic outcomes. This literature review will therefore focus specifically on disparities within the U.S. healthcare system. Ensuring equitable access to these evidence-based therapies is not only essential to align with contemporary clinical guidelines but also a critical opportunity to reduce preventable cardiovascular and renal complications while advancing equity in chronic disease management. Addressing the structural and systemic drivers underlying these disparities is fundamental to delivering truly patient-centered diabetes care.

## Review

Search strategy

This study was conducted as a narrative review aimed at synthesizing current evidence on racial and socioeconomic disparities in the utilization of SGLT2 inhibitors and GLP-1 receptor agonists, with a particular focus on patients with type 2 diabetes and cardiovascular disease in the United States. A comprehensive literature search was performed using PubMed and Google Scholar, incorporating combinations of keywords and Medical Subject Headings (MeSH) terms including “SGLT2 inhibitors,” “GLP-1 receptor agonists,” “type 2 diabetes,” “cardiovascular disease,” “racial disparities,” “health disparities,” “healthcare access,” “socioeconomic factors,” “prescribing patterns,” and “utilization,” with Boolean operators applied to refine results. Studies published between 2010 and 2025 were included to capture contemporary evidence following the introduction and widespread use of these therapies. Eligible studies included original research articles, observational cohort studies, and systematic reviews that evaluated disparities in prescribing or access to SGLT2 inhibitors and/or GLP-1 receptor agonists among adults with type 2 diabetes, particularly within U.S. populations. Studies focusing exclusively on type 1 diabetes, non-English publications, case reports, editorials, or those lacking sufficient methodological detail were excluded. Titles and abstracts were initially screened for relevance, followed by full-text review to determine eligibility based on predefined inclusion and exclusion criteria, and the most relevant studies were selected for inclusion in this review (Table 1).

**Table 1:** Summary of Key Studies Examining Disparities in Cardiometabolic Therapies SGLT2is: Sodium-glucose cotransporter-2 inhibitors, GLP-1 RAs: Glucagon-like peptide-1 receptor agonists, T2DM: Type 2 diabetes, CVD: Cardiovascular disease.

Authorship & Year	Objective	Conclusion
Brown et al., 2021 [1]	To review the emerging and established indications for SGLT2 inhibitors (SGLT2is) and GLP-1 receptor agonists (GLP-1 RAs), spotlighting their roles beyond glucose lowering.	SGLT2is and GLP-1 RAs have both demonstrated benefits that are beyond glycemic control, i.e., reducing risk of cardiovascular events, overall mortality, and even hospitalizations for heart failure.
McCoy et al., 2020 [2]	To investigate multimorbidity and glycemic control among diabetics.	Glycemic control strategies often ignore individual risk and multimorbidity.
Myasoedova et al., 2024 [3]	To explore the anti-inflammatory and antioxidant properties of SGLT2is and GLP-1 RAs, and to elucidate how these mechanisms contribute to the cardiovascular protective effects observed with these therapies in patients with type 2 diabetes mellitus (T2DM).	SGLT2is and GLP-1 RAs provide cardiovascular protection in T2DM by reducing oxidative stress and inflammation, offering benefits beyond glycemic control and supporting comprehensive diabetes management.
Hill-Briggs et al., 2020 [4]	To examine the association between diabetes risk and the social determinants of health (specifically, the five domains: socioeconomic status, neighborhood and physical environment, food environment, health care, and social context).	There is strong, consistent evidence that the five domains impact diabetes risk, disease management, and complication rates, and that racial and ethnic minorities/low-income communities experience disproportionate burdens of diabetes incidence and mortality.
Batchelor et al., 2021 [5]	To study site diversity on cardiology trial performance.	Sites with diverse subjects yield more representative data and performance.
Yedjou et al., 2024 [6]	To examine racial disparities in diabetes mellitus among ethnic groups in the U.S.	African Americans, Hispanics, and Native Americans exhibit notably higher rates of diabetes compared to non-Hispanic Whites and Asians, notably driven by socioeconomic factors such as limited education, as well as income.
Zheng et al., 2024 [7]	To survey advancements involving GLP-1 RAs, provide a comprehensive update on the physiological role in glucose regulation, and explore the clinical applications of this medication.	GLP-1 RAs exert varied metabolic and cellular effects aside from glycemic control, which include the enhancement of insulin release and inhibition of glucagon secretion.
Vallon & Thomson, 2017 [8]	To investigate racial disparities in the use of glucose monitors as well as insulin pumps in adults with type 1 diabetes.	There are significant disparities in the access and use of technology between Black and non-Black individuals with type 1 diabetes. Black patients were less likely to use pumps or glucose monitors.
Crisci & Salzano, 2024 [9]	To evaluate regional/ethnic differences in SGLT2i impact in heart failure, focus on Asia.	Asian populations receive less SGLT2i use despite the cardiovascular benefits.
Davies et al., 2022 [10]	To provide an updated and more patient-centered framework for managing hyperglycemia in adults with T2DM.	Managing hyperglycemia in T2DM requires a holistic strategy that goes beyond glucose-centric approaches. Lifestyle modification and focusing on the social determinants of health remain the cornerstone of said management.
Kunutsor et al., 2024 [11]	To conduct a meta-analysis of literature related to ethnic differences in GLP-1RA/SGLT2i outcomes.	Regional and ethnic outcome variability suggests inequitable therapy distribution.
Piya & Hocking, 2024 [12]	To discuss challenges to medication access in ethnic groups.	Ethnic minorities face major barriers to accessing advanced diabetes therapies.
Cai et al., 2022 [13]	To quantify inequities in diabetes pharmacotherapy by race/ethnicity.	Black and Hispanic patients receive newer treatments less often than their white and non-Hispanic counterparts.
Morris et al., 2021 [14]	To analyze racial differences in heart failure treatment with SGLT2is.	SGLT2is are underutilized in Black populations despite need.
Ezzatvar et al., 2021 [15]	To quantify the racial and ethnic differences in the risk of diabetic complications as well as all-cause mortality.	There is a notable racial and ethnic disparity in the long-term outcomes of patients with diabetes. Black individuals faced a 54% increased risk of end-stage renal disease.
Ding & Glied, 2022 [16]	To examine trends and disparities in use of and expenditures for three new classes of diabetes drugs (glucagon-like peptide-1 receptor agonist, dipeptidyl peptidase 4 inhibitors, and sodium-glucose cotransporter-2 inhibitors) by race, ethnicity, insurance coverage, education, and income.	Drug usage rates for Latinx/Hispanic people fall midway between rates for white and Black people. Similar disparities by insurance coverage also emerged shortly after the drugs became available and continue today. In addition, lower socioeconomic status has been independently associated with lower uptake of new medications.
Vasti et al., 2023 [17]	To evaluate the adoption disparities of new diabetes therapies with cardiovascular benefits.	Racial and ethnic minorities are less likely to receive these therapies.
Rodriguez et al., 2024 [18]	To examine pharmacy-level disparities in SGLT2i/GLP-1RA dispensing.	Disparities in drug dispensing patterns across ethnic groups.
Nye, 2024 [19]	To report disparity in prescribing SGLT2is to minority groups.	Black and Asian patients are prescribed SGLT2 inhibitors less often than their white counterparts.
Schaffer, 2021 [20]	To highlight gender and race disparities in SGLT2i prescription.	Women and minorities are less frequently prescribed SGLT2is.
LeBlanc, 2024 [21]	Explores how sex and gender discrepancies affect cardiovascular disease treatment and risk outcomes in those with type 2 diabetes. More specifically focused on identifying female-specific CVD risk factors and evaluating them as limitations in trials regarding sex specific data for T2DM medications, including SGLT-2i	Improving outcomes for women with T2DM requires sex-specific cardiovascular risk factors that should be taken into consideration when screening and making treatment decisions
Tan et al., 2024 [22]	To evaluate the effectiveness of various GLP-1 receptor agonists vs. dipeptidyl peptidase-4 inhibitors on glycemic control as well as weight over 1 year in patients with T2DM.	GLP-1 receptor agonists significantly outperformed dipeptidyl peptidase‑4 inhibitors, improving both HbA1c and weight amongst those with T2DM.
Badve et al., 2024 [23]	To evaluate the effects of GLP-1 RAs on renal and cardiovascular outcomes by conducting a meta-analysis of data from randomized controlled trials involving patients with T2DM and/or high cardiometabolic risk.	GLP-1 receptor agonists significantly reduce the risk of major adverse cardiovascular events (MACE) and may confer renal protective benefits, especially in individuals with existing cardiovascular or kidney disease. These findings support the broader use of GLP-1 RAs as part of integrated management strategies for cardiometabolic diseases.
Pihoker et al., 2023 [24]	To examine health care coverage, access to care, and frequency of care in those with type 1 and type 2 diabetes in the U.S., and determine how those factors are associated with glycemic control.	Patients with type 1 and type 2 diabetes consistently reported lower rates of health care coverage, limited access to usual care providers, and less frequent diabetes care usage.
Eberly et al., 2021 [25]	Link SGLT2i use with demographic and socioeconomic factors.	Lower use among minorities and lower socioeconomic groups.
Hwang et al., 2025 [26]	To examine the fiscal and health impact of expanding medicare coverage to include GLP-1 RAs under medicare Part D	Expanding Medicare Part D to include GLP-1 RAs leads to significant health gains, and more notably, a reduction in cardiovascular events. Still, it comes with a notable increase in the burden on Medicare’s budget.
Jack et al., 2025 [27]	To look at disparities in the prescription as well as utilization of SGLT2is for cardiorenal protection amongst patients with and without T2DM, with a focus on ethnicity, race, and socioeconomic inequity.	There are persistent disparities in SGLT2i use across ethnic and racial groups and more particularly amongst underserved populations.
Bolden, 2024 [28]	To evaluate patterns of evidence-based prescription of SGLT2is and GLP-1 RAs over time and by race in U.S. Veterans with T2DM and coronary artery disease.	Prescription of SGLT2i did not differ by race, but Black patients were less likely than White patients to have evidence-based prescription of a GLP-1 RA. Disparities in evidence-based use of cardiovascular treatments may be specific to certain drug classes, even in a healthcare system with few economic barriers.
Wang et al., 2025 [29]	To assess uptake disparities of SGLT2is and GLP-1 RAs among Medicare patients.	Marked disparities exist in usage among racial/ethnic groups.
Oliver et al., 2024 [30]	To analyze GLP-1 RA and SGLT2i use by race in the U.S. military health system (MHS).	Significant racial disparities exist within the MHS in drug use.
Kani et al., 2025 [31]	To conduct a systematic review of SGLT2i efficacy by race and region.	Regional and racial variations affect efficacy and outcomes.
Hassan et al., 2023 [32]	To examine racial disparities in diabetes prevalence and management.	Structural barriers hinder optimal care among minorities.

Mechanisms of action and clinical benefits

GLP-1 Receptor Agonists

Glucagon-like peptide-1 receptor agonists (GLP-1 RAs) are a class of antidiabetic therapies that mimic the actions of endogenous GLP-1, enhancing glucose-dependent insulin secretion while suppressing inappropriate glucagon release. By reducing hepatic glucose output and improving post-prandial insulin response, these agents help restore more physiologic glycemic control in patients with type 2 diabetes. GLP-1 RAs also slow gastric emptying and promote satiety, leading to clinically meaningful weight loss, an effect particularly advantageous in patients with obesity-related insulin resistance. Unlike many traditional glucose-lowering therapies, GLP-1 RAs therefore combine glycemic benefit with weight reduction, aligning closely with modern cardiometabolic treatment goals [6].

Beyond metabolic control, GLP-1 RAs have demonstrated compelling cardiovascular benefit. Large cardiovascular outcome trials, including LEADER (Liraglutide Effect and Action in Diabetes: Evaluation of Cardiovascular Outcome Results), and AMPLITUDE-O (Effect of Efpeglenatide on Cardiovascular Outcomes), among others, consistently show reductions in major adverse cardiovascular events, particularly myocardial infarction, stroke, and cardiovascular mortality, among patients with established atherosclerotic cardiovascular disease or at high risk for cardiovascular disease (CVD). These effects appear to extend beyond glucose-lowering alone, likely reflecting favorable actions on inflammation, endothelial function, body weight, and blood pressure. As a result, current professional guidelines recommend GLP-1 RAs as first-line cardioprotective glucose-lowering therapy for patients with type 2 diabetes and established or high-risk coronary artery disease - independent of baseline glycated hemoglobin (HbA1c) or background metformin use. Taken together, the evidence base supporting GLP-1 RAs is both robust and clinically meaningful: these agents safely improve glycemic control, promote weight loss, and critically reduce the burden of coronary and cerebrovascular events in patients already facing heightened cardiovascular risk [7]

SGLT2 Inhibitors

Sodium-glucose co-transporter-2 inhibitors (SGLT2is) are a relatively new class of oral antihyperglycemic agents that lower plasma glucose through a renal mechanism. By selectively inhibiting SGLT-2 transporters in the proximal renal tubule, the primary site of filtered glucose reabsorption, these agents promote glucosuria and thereby reduce circulating glucose levels in a largely insulin-independent manner. This mechanism not only improves glycemic control but also confers several metabolic benefits, including modest weight loss from urinary calorie loss and mild natriuresis leading to reductions in systolic and diastolic blood pressure [8].

More importantly, SGLT2 inhibitors have redefined the contemporary management of cardiovascular and renal disease in patients with type 2 diabetes. Early cardiovascular outcome trials consistently demonstrated significant reductions in heart failure hospitalizations and improved renal outcomes, with benefits observed irrespective of baseline atherosclerotic cardiovascular disease status. This paradigm shift was further solidified by dedicated heart failure trials such as DAPA-HF and EMPEROR-Reduced, which showed meaningful reductions in cardiovascular death and heart failure hospitalizations even among patients without diabetes, establishing a clear class effect [9]. These benefits are thought to be mediated through improvements in myocardial energetics, reductions in preload and afterload, and favorable effects on ventricular remodeling. While the most consistent and pronounced effects are seen in heart failure and renal protection, meta-analyses also suggest modest reductions in major adverse cardiovascular events, particularly in patients with established coronary artery disease [9].

Because of this robust and reproducible evidence base, contemporary cardiology and endocrine guidelines now recommend SGLT2is as foundational therapy for patients with type 2 diabetes and high cardiovascular or renal risk, and as core therapy for heart failure across the ejection-fraction spectrum, independent of glycemic status [9,10]. These agents therefore represent not only glucose-lowering medications, but truly cardiorenal protective therapies, helping to reduce hospitalization, disease progression, and long-term cardiovascular burden in patients at risk for or already living with coronary artery disease [10].

Disparities in prescription patterns

Racial and Ethnic Disparities

Diabetes mellitus affects more than 537 million adults worldwide and is projected to reach nearly 783 million by 2045, with substantial regional, racial, and socioeconomic variation in both disease burden and access to care [11,12]. Because type 2 diabetes is tightly linked to atherosclerotic cardiovascular disease, particularly coronary artery disease (CAD), inequities in diabetes management directly contribute to disparities in cardiovascular outcomes [13].

Accumulating evidence demonstrates that GLP-1 receptor agonists (GLP-1 RAs) and SGLT2 inhibitors (SGLT2is) are prescribed less frequently to racial and ethnic minority populations despite their established cardioprotective benefits. Analyses from large healthcare systems, including the Veterans Health Administration, show that Black patients are significantly less likely than White patients to receive these therapies [13]. This gap is especially concerning given the higher prevalence of T2DM, heart failure, chronic kidney disease, and premature CAD among Black individuals, along with worse cardiovascular outcomes [14,15]. Similar underutilization has been documented among Hispanic patients compared with non-Hispanic populations [16].

Notably, these disparities persist even within integrated healthcare systems that reduce financial barriers, suggesting that cost alone does not fully explain unequal prescribing [17]. Structural factors, including implicit provider bias, variation in clinician familiarity with cardiometabolic therapies, limited access to specialty care, and communication barriers related to language and health literacy, further contribute to inequitable use [18,19].

Given that GLP-1 RAs and SGLT2is reduce major adverse cardiovascular events, slow kidney disease progression, and lower heart failure hospitalizations, failure to equitably deploy these agents represents a missed opportunity to reduce disparities in CAD-related morbidity and mortality. Addressing these gaps through clinician education, standardized treatment pathways, culturally responsive care models, and supportive policy reforms is essential to advancing cardiovascular equity.

Gender Disparities

A growing body of evidence indicates that women with type 2 diabetes mellitus are less likely than men to receive prescriptions for GLP-1 receptor agonists and SGLT2 inhibitors, even when clinical indications are comparable. Several interrelated factors appear to contribute to this treatment gap. Implicit bias during clinical risk assessment may lead to systematic underestimation of cardiovascular risk in women, along with assumptions regarding lower adherence to therapy. In addition, women often present with atypical or non-classic manifestations of cardiovascular disease, which may delay recognition of cardiometabolic risk and attenuate the urgency of initiating advanced therapies. Differences in healthcare-seeking behavior, including prioritization of family needs and postponement of personal care, further compound these challenges. Collectively, these factors contribute to persistent gender-based disparities in optimal diabetes and cardiovascular management, underscoring the need for gender-sensitive approaches to risk stratification and treatment selection [20,21].

Socioeconomic Disparities

Socioeconomic status is a major determinant of access to GLP-1 RAs and SGLT2 inhibitors. Numerous studies demonstrate lower utilization of these agents among individuals with lower income, limited educational attainment, and inadequate insurance coverage. High out-of-pocket costs, including deductibles, copayments, and formulary restrictions, remain substantial barriers even for insured patients. These financial pressures not only discourage treatment initiation but also contribute to poor adherence and early discontinuation, thereby diminishing potential cardiometabolic benefit [6].

Patients from socioeconomically disadvantaged backgrounds are also more likely to receive care in resource-limited settings, where clinician familiarity with newer cardioprotective diabetes therapies may be variable. Moreover, social determinants of health - such as housing instability, food insecurity, and limited transportation - further impede continuity of care and medication access. Because these populations often carry the highest burden of cardiometabolic disease, failure to overcome socioeconomic barriers perpetuates preventable disparities in outcomes. Addressing these gaps will require coordinated policy initiatives, insurance reforms, clinician education, and system-level strategies to improve affordability and access [22].

Clinical Indications and Comorbidities

Current evidence strongly supports the use of GLP-1 receptor agonists and SGLT2 inhibitors in patients with type 2 diabetes and established cardiovascular disease, chronic kidney disease, or obesity, independent of baseline glycemic control [1]. These comorbidities confer markedly elevated risk for adverse cardiovascular events, and advanced cardiometabolic therapies offer substantial protective benefit. However, disparities in prescribing persist even among patients who clearly meet guideline-based indications. For example, Black patients who qualify for SGLT2 inhibitor therapy are significantly less likely to receive these medications than White patients with similar clinical profiles [6].

Such findings indicate that unequal utilization cannot be explained by clinical factors alone and instead reflects broader structural and systemic barriers. Implementation of standardized, guideline-driven decision support tools, targeted clinician education, and equity-focused quality metrics may help ensure that all eligible patients receive evidence-based cardioprotective therapies.

Contributing factors to disparities

Healthcare Provider Factors

Several provider-related factors contribute to persistent disparities in the prescribing of GLP-1 receptor agonists and SGLT2 inhibitors despite strong evidence supporting their cardiometabolic benefits. Implicit bias remains a central concern, influencing clinical judgment in ways that may lead to underestimation of cardiometabolic risk or assumptions about medication adherence, particularly among racial and ethnic minority and socioeconomically disadvantaged patients. Such biases can result in fewer discussions about, and lower rates of initiation of, advanced cardiometabolic therapies [23].

Knowledge gaps also play an important role. Diabetes treatment guidelines evolve rapidly, and not all clinicians, particularly those practicing in resource-limited or rural settings, are fully aware of recent evidence supporting the cardiovascular and renal benefits of GLP-1 RAs and SGLT2is. Some providers continue to rely on traditional glucose-centric treatment approaches rather than contemporary cardiometabolic frameworks, reflecting limited access to continuing medical education or specialty support.

Time constraints further compound these challenges. High clinical workloads and brief outpatient encounters restrict opportunities for detailed counseling regarding the mechanisms, benefits, risks, and costs of newer therapies. In busy practices, particularly when caring for patients with multiple comorbidities or language barriers, clinicians may default to familiar or simpler regimens rather than initiating evidence-based but more complex treatment strategies.

Patient Factors

Health Literacy: Limited health literacy significantly impedes equitable access to GLP-1 RAs and SGLT2is. Patients with lower health literacy may struggle to understand the progressive nature of diabetes, its cardiovascular implications, and the long-term benefits of newer therapies, leading to uncertainty, fear of adverse effects, and reluctance to initiate treatment. Older adults, individuals with limited formal education, and those with limited proficiency in the dominant language are disproportionately affected [24].

Cultural Beliefs and Language Barriers: Cultural perceptions of illness and treatment influence healthcare engagement and acceptance of novel therapies. In some communities, skepticism toward pharmaceutical interventions or preference for traditional healing practices may reduce willingness to initiate newer, high-cost medications. Language discordance between patients and clinicians can further impair understanding of treatment rationale and shared decision-making. Use of trained medical interpreters and culturally tailored educational materials is essential to building trust and improving uptake of evidence-based therapies [25].

Financial Constraints: High medication costs remain among the most formidable barriers to GLP-1 RA and SGLT2i use. These agents are substantially more expensive than many generic glucose-lowering therapies, and patients frequently encounter formulary restrictions, prior authorization requirements, and high out-of-pocket expenses. Financial strain may force patients to delay initiation, ration doses, or discontinue therapy altogether, particularly when faced with competing necessities such as housing, food, and transportation. Addressing these barriers will require system-level solutions, including insurance reforms, patient assistance programs, and policies aimed at improving medication affordability [26].

Systemic Factors

Healthcare Access: Access to specialty care and modern healthcare infrastructure is a major determinant of whether patients receive GLP-1 receptor agonists and SGLT2 inhibitors. Clinicians practicing in rural and underserved urban settings often have limited exposure to rapidly evolving diabetes treatment guidelines and may lack ready access to endocrinology or cardiology subspecialty support. Consequently, patients in these regions are more likely to receive care from general practitioners rather than specialists familiar with contemporary cardiometabolic therapies. Structural barriers, including transportation challenges, limited clinic availability, long wait times, and workforce shortages, further restrict access to advanced care. These obstacles disproportionately affect low-income individuals, people with disabilities, and racial and ethnic minority populations, thereby reinforcing existing inequities in diabetes and cardiovascular outcomes [6].

Insurance Policies: Insurance-related barriers represent one of the most significant structural impediments to GLP-1 RA and SGLT2i use. Many insurers place these agents on higher formulary tiers, resulting in substantial out-of-pocket costs even for insured patients. Prior authorization requirements add additional administrative burden and delay therapy initiation, discouraging both clinicians and patients from pursuing these evidence-based treatments. Ironically, patients with multiple comorbidities, who stand to benefit most from the cardiorenal protective effects of these medications, often encounter the greatest difficulty obtaining timely access [10].

Clinical Guidelines and Implementation: Although major professional societies endorse GLP-1 RAs and SGLT2 inhibitors for patients with type 2 diabetes and high cardiovascular or renal risk, translation of guidelines into routine practice remains inconsistent [10]. Variability in interpretation, limited dissemination, and insufficient continuing medical education contribute to uneven adoption across clinical settings. Furthermore, existing guidelines rarely address how social, racial, and economic factors influence real-world access to therapy, leaving clinicians without practical frameworks to mitigate disparities [6]. Development of clearer initiation algorithms, integration of decision-support tools into electronic health records, and targeted implementation strategies across diverse practice environments are essential to promote equitable delivery of cardiometabolic care.

Strategies to address disparities

Provider Education and Training

Improving clinician knowledge of the cardiometabolic benefits of GLP-1 receptor agonists (GLP-1 RAs) and SGLT2 inhibitors (SGLT2is) is a foundational step toward reducing inequities in diabetes care. Despite strong evidence demonstrating reductions in major adverse cardiovascular events, heart failure hospitalization, and progression of chronic kidney disease, many clinicians remain unfamiliar with the full scope of benefits or optimal patient selection [10]. Expansion of continuing medical education programs focused on contemporary guidelines, trial evidence, and practical prescribing strategies can facilitate more consistent, evidence-based use.

Equally important is formal training in implicit bias recognition and cultural competency, as unconscious assumptions may influence risk assessment, expectations of adherence, and therapeutic recommendations. Integrating bias-awareness and equity-focused curricula into professional development improves clinicians’ ability to deliver fair, individualized care that aligns clinical evidence with patient context [6].

Patient Engagement and Support

Effective patient engagement is essential to improving uptake and adherence to cardioprotective diabetes therapies. Culturally responsive education that clearly explains how GLP-1 RAs and SGLT2 inhibitors protect the heart and kidneys can empower patients to participate meaningfully in shared decision-making. Community health workers and patient navigators play a critical role in bridging healthcare systems with underserved communities by providing tailored education, assisting with appointment scheduling, and connecting patients to insurance and medication-assistance programs [27]. These approaches strengthen trust, enhance communication, and improve continuity of care.

Evidence That Disparities Persist Across Health Systems

Persistent inequities in prescribing have been documented across multiple healthcare environments. In the Veterans Health Administration, where financial barriers are minimized, Black patients remain less likely than White patients to receive GLP-1 RAs and SGLT2is, suggesting that factors beyond cost, such as clinician decision-making and knowledge gaps, contribute to disparities [28]. In a large commercially insured population, race and ethnicity, sex, and socioeconomic status were independently associated with lower use of SGLT2is, with particularly reduced access among Black, Hispanic, and Asian patients despite their high burden of atherosclerotic cardiovascular disease [25].

Similarly, Medicare analyses from 2013-2019 demonstrate persistent racial and ethnic disparities in use of GLP-1 RAs and SGLT2is across comorbid conditions, including heart failure and chronic kidney disease [29]. Data from the U.S. Military Health System, an environment with relatively uniform access, also reveal continued racial differences in utilization, reinforcing the influence of structural and behavioral determinants of care [30,31]. Collectively, these findings underscore a consistent gap between evidence-based recommendations and real-world practice, particularly among patients with multiple chronic conditions who stand to benefit most from these therapies [2,33].

Policy and System-Level Interventions

Insurance reforms are essential to reduce patient cost-sharing and expand coverage for GLP-1 receptor agonists and SGLT2 inhibitors, thereby improving affordability and access to these evidence-based therapies. Integrating clinical decision-support tools within electronic health records can further enhance utilization by prompting clinicians to identify eligible patients and standardizing prescribing practices. In addition, implementing quality improvement initiatives, including equity-focused performance metrics and regular prescribing audits, can promote accountability and drive sustained improvements in the equitable delivery of these cardiometabolic therapies [27].

Efforts to reduce health disparities include increasing representation in National Institutes of Health-funded research to 40% by 2030, investing in research infrastructure in underserved communities, and strengthening community engagement and implementation of findings. However, financial barriers remain a major obstacle. Medication costs, out-of-pocket expenses, and insurance type continue to influence access to SGLT2 inhibitors and GLP-1 receptor agonists [27]. In a study of over 3,000 adults with type 2 diabetes from the 2017-2018 Medical Expenditure Panel Survey, Medicare beneficiaries were less likely to receive these therapies compared with those with private insurance (OR 0.60; 95% CI, 0.36-0.99). Notably, race and ethnicity explained about 26% of the variation, highlighting the ongoing impact of structural and financial inequities [27].

## Conclusions

Despite compelling evidence demonstrating robust cardiovascular and renal protection, the use of SGLT2 inhibitors and GLP-1 receptor agonists remains uneven across racial and ethnic groups. These therapies are disproportionately underutilized among racial and ethnic minority populations, women, and individuals from socioeconomically disadvantaged backgrounds, limiting their ability to mitigate cardiovascular risk and improve long-term cardiometabolic outcomes.

Such inequities reinforce existing disparities in cardiovascular disease burden and hinder the translation of landmark clinical trial evidence into real-world benefit. Closing this gap will require a coordinated, multi-level strategy encompassing clinician education, recognition and mitigation of implicit bias, and system-wide reforms. Healthcare systems must adopt data-driven and equity-focused implementation strategies to ensure that GLP-1 receptor agonists and SGLT2 inhibitors are prescribed safely, consistently, and equitably to all eligible patients. Addressing clinical, structural, and social determinants in parallel is essential to realizing the full population-level cardiovascular benefit of these therapies and advancing equity in modern diabetes and cardiovascular care.
